# Antagonism of Pulmonary Phthisis and of Intermittent Fever

**Published:** 1844-10

**Authors:** 


*Antagonism of Pulmonary Phthisis and of Intermittent Fever.*—At the scientific congress of Lucca this subject was warmly discussed, without any satisfactory result having been

---

arrived at. Dr. Salvagnoli presenting a series of cases, accompanied by a statistical table, tending to show that in the Maremmi district of Tuscany, where intermittent fevers are very numerous, pulmonary phthisis and scrofula is but rarely met with. This pathological fact he endeavors to explain in the following manner:—If we admit (says he) a physiological antagonism between the liver and the lungs, which most physiologist do, it is easy to understand why phthisis should be rare where intermittent fever is common. Malaria acts principally on the abdominal viscera, increasing their action and volume. This increase of abdominal vitality diminishes the vital activity of the lungs, and thus prevents the development of tubercles in their tissue.

Dr. Renzi criticised Dr. Salvagnoli's statistical table, and remarked that in marshy districts the number of individuals who arrive at a favorable age for the development of pulmonary phthisis is relatively smaller than in more favored localities, and that consequently a calculation established only on the number of deaths from phthisis in such localities could not be exact. If the generations disappear during childhood,—if the medium age, which in towns is thirty years, is infinitely less in marshy districts, it becomes evident that more persons will die from the inflammatory diseases of early life, and fewer from chronic affections.

Dr. Griffa confirmed the observations of Dr. Salvagnoli respecting the rarity of phthisis in the Maremmi. Dr. Rino, on the contrary, maintained from observations made at the Clinique of Pisa, that phthisis was common in marshy countries. These views were also supported by Dr. Tuchetti, who asserted that in the marshy districts of Fucecchio and Bientina he had not remarked the antagonism, which was described by Dr. Salvagnoli as existing in the Maremmi. Dr. Trompeo stated that he had great opportunities for observation in many localities in Piedmont, where malaria was kept up by the rice-grounds, and in Sardinia, where intermittents are extremely frequent and severe; and that both these countries he had found phthisis very rare wherever intermittent fever was common.

